# Treatment adherence in chronic myeloid leukaemia patients receiving tyrosine kinase inhibitors

**DOI:** 10.1007/s12032-017-0958-6

**Published:** 2017-04-25

**Authors:** Anna Rychter, Piotr Jerzmanowski, Adam Hołub, Zofia Specht-Szwoch, Violetta Kalinowska, Urszula Tęgowska, Ilona Seferyńska, Agnieszka Kołkowska-Leśniak, Ewa Lech-Marańda, Joanna Góra-Tybor

**Affiliations:** 1 Department of Hematology, Medical University of Lodz, 2 Ciolkowskiego Street, 93-510 Lodz, Poland; 2 Hematology Clinic, Multidisciplinary Center for Oncology and Traumatology, Lodz, Poland; 3 Department of Hematology, Centre of Oncology, Gdańsk, Poland; 4 Department of Hematology, Copernicus Memorial Hospital, Toruń, Poland; 5 Department of Hematology, Institute of Hematology and Transfusion Medicine, Warsaw, Poland

**Keywords:** Chronic myeloid leukaemia, Tyrosine kinase inhibitor, Imatinib, Dasatinib, Nilotinib, Adherence

## Abstract

Failure to comply with treatment recommendations is very common in patients, but still poorly recognised by doctors. The current practice of using oral therapy on a large scale has been increasingly adopted for cancer patients. Chronic myeloid leukaemia (CML) is just such an example, where the introduction of taking new oral medications, the tyrosine kinase BCR-ABL inhibitors (TKI), has now revolutionised the treatment. The aim of our study was to assess treatment adherence in a group of Polish CML patients (a survey was conducted on 140 patient aged ≥18 years) treated with oral TKI (imatinib, dasatinib and nilotinib) taking into account the following variables: gender, age, education, place of residence, family circumstances and duration of therapy. In addition, we evaluated whether there is a relationship between how patients perceive their level of adherence to treatment recommendations with how subjectively the required dosage regimen was followed. Half the patients admitted to skipping at least one drug dose during the entire course of treatment and 39% did so within their last treatment month. Patients were also found to overestimate their own adherence assessment; around 60% of those missing at least 1 drug dose within the last treatment month believed they ‘always’ followed recommendations. The study demonstrated that adherence deteriorates over time. Furthermore, patients aged >65 years and patients suffering at least one comorbid disease had better adherence (*p* < 0.011). There were no differences in adherence among patients treated with imatinib, dasatinib and nilotinib (*p* = 0.249).

## Introduction

Chronic myeloid leukaemia (CML) is myeloproliferative neoplasm constituting around 15% of newly diagnosed leukaemia in adult patients [1]. A key feature of this disease is the presence of the Philadelphia Chromosome (Ph) resulting from reciprocal translocation between chromosomes 9 and 22 that causes the Abelson gene to fuse with the breakpoint cluster region of the BCR gene. This translocation creates two novel genes: *BCR*-*ABL* on chromosome 22 (chromosome Ph) and *ABL*-*BCR* gene on chromosome 9. The former encodes a 210-kD protein exhibiting abnormally increased tyrosine kinase activity (TK). By recognising this key role of the *BCR*-*ABL* gene in CML development, it has thus been possible to devise tyrosine kinase inhibitors (TKI), that block ATP attachment, thereby inhibiting this enzyme’s activity [2–4].

In 1998, the first TKI introduced into clinical practice was imatinib (IM; Glivec^®^, Novartis Pharmaceuticals). Because of its high efficacy and low toxicity, this therapy became first choice for CML patients [5–8]. In subsequent years, new and so-called second generation TKI inhibitors have appeared; dasatinib (Sprycel^®^, Bristol-Myers Squibb) and nilotinib (Tasigna^®^, Novartis Pharmaceuticals), and then bosutinib (Bosulif^®^, Pfizer) [9–11]. These were originally used for second-line treatment in cases of intolerance to, or ineffectiveness of IM therapy; they have subsequently also been registered for first-line treatment (dasatinib and nilotinib) [12–15]. Introducing TKI therapy for CML has revolutionised treatment of this disease. Before the TKI era, overall survival (OS) of CML patients was about 3–4 years [16], whereas the currently achieved OS is now similar to the general population, leading to CML becoming considered a chronic disease [9, 17–20].

All TKIs are oral medications, taken on one’s own by the patient. To obtain an optimal effect of treatment, thus requires patient adherence, i.e. taking the correct dose regularly, at appropriate intervals and at the right time in relation to meals [21, 22].

Non-adherence is, however, very common in patients with chronic diseases receiving oral medications. In research studies, the extent of adherence in patients suffering from diabetes, hypertension and coronary artery disease has been shown to range from 20 to 40%, whilst most studies consider ‘optimal adherence’ to be at 80–95% levels of the recommended dose [23]. The rise of cancer therapies administered orally is coupled with poor adherence for increasing numbers of cancer patients. Only 14.2% of CML patients were found in one study to adhere 100% with IM dose regimen, with 71% taking less than the stipulated dose and 14.8% taking higher doses [24]. It has also been shown that poor adherence is associated with sub-optimal responses to therapy, where therapeutic goals, like complete cytogenetic response (CCyR) and major molecular response (MMoR), are not achieved [21, 22]. Furthermore, the failure to adhere to treatment recommendations is frequently underestimated by doctors. In cases of CML, the lack of efficacy for the first-generation IM drives the changing of therapy to using much more expensive and more toxic TKIs of the second generation. Therefore, diagnosing the problem of ‘adherence’ by medical staff is essential to successful treatment.

The study aim was to evaluate the systematic use of TKIs (IM, dasatinib and nilotinib) for CML patients and to determine the impact of various factors such as demographics (gender, age, education, place of residence, family situation), the coexistence of other diseases, TKI side effect rates and the duration of therapy on patients’ adherence to treatment recommendations. Patients’ self-assessment was also compared with their declared rates for omitting medication doses.

## Materials and methods

The basic study tools were questionnaires completed by patients with CML in its chronic phase aged ≥18 years treated with TKIs for at least 6 months, who were under observation at four selected haematological centres in Poland from Lodz, Torun, Gdansk and Warsaw. The study was conducted anonymously and voluntarily. Each patient was fully informed about the study purpose and conduct (i.e. on how the questionnaires should be filled out), with the choice of opting out at anytime during the study. All patients participating in the study provided written informed consent. Those excluded were on the basis of any intellectual disability that prevented understanding the questions within the questionnaire or/if psychiatric disorders were present.

Questionnaires, prepared in Polish language, were based on the diagnostic survey method and consisted of 17 closed questions which the patients replied to at their follow-up doctor’s appointments. These were on socio-demographics (gender, age, education, place of residence, family situation) and ones designed to evaluate non-adherence levels in taking medication and a subjective assessment of their adherence to following treatment recommendations. Questions included those on how many doses were skipped within the last month prior to the visit as well as over the entire treatment period. In addition, questions also asked for the reasons why doses were skipped, on comorbidities, adequacy of disease information received from the doctor and their own assessment of treatment adherence. For the latter, the patient’s attitude/agreement was assessed on a five-point Likert scale (from 1, ‘does not comply’ to 5, ‘always complies’) which the patient replied to at their next visit. Confidentiality of replies was assured through using sealed envelopes. Because all TKI treatments in Poland are publically financed (depending on the type of treatment) by the National Health Fund, any economic factors were excluded from this study. Ethical approval for the study was received by the Bioethics Committee at the Medical University of Lodz (No. RNN/226/15/EC).

### Statistical analysis

The Shapiro–Wilk test was used to test interval and ordinal data for normality. The significance of intergroup differences was assessed by the Mann–Whitney–Wilcoxon *U* test and the Student’s *t* test or by ANOVA or the Kruskall–Wallis test, as appropriate, depending on the variable distribution. The relationships between quantitative variables were determined by Spearman’s rho correlation coefficient. Results were expressed as mean and standard deviation, or as numbers and percentages of the analysed subgroups. Differences in categorical data were assessed by the Chi-square test or Fisher’s exact test. Multivariate analysis was performed by stepwise logistic regression using forward selections of a model at <0.1 significance. The goodness of fit was verified by the Hosmer–Lemeshow test. Null hypothesis were rejected at the *p* < 0.05 level. All statistics were performed using Statistica software.

## Results

### Patient population

Subjects were 153 patients with CML in chronic phase treated with TKI, of whom 140 replied to all questions set by the study. There were 70 men and 70 women. Imatinib was administered to 101 patients, 25 received dasatinib, and 14 were given nilotinib. Patient details are shown in Table 1.

**Table 1:** Study group characteristics (patients aged ≥18 years diagnosed with CML and treated with imatinib, dasatinib and nilotinib; *n* = 140)

Variable	*n* (%)
Gender	
Women	70 (50)
Men	70 (50)
Age	
<30	7 (5)
30–65	96 (69)
>65	37 (26)
Education	
Basic	43 (31)
Secondary	58 (41)
Higher	39 (28)
Place of residence	
Countryside	51 (36)
Town/city	89 (64)
Family circumstances	
Single	22 (16)
Living with family	118 (84)
Comorbidities	
None	51 (36)
At least 1	89 (64)
Adverse effects	
None	33 (24)
At least 1	107 (76)
TKI	
Imatinib	101 (72)
Dasatinib	25 (18)
Nilotinib	14 (10)

### Skipping TKI doses in the month prior to follow-up doctor’s appointment

Within the 140 study group, two subjects did not reply as to whether they skipped medication doses in the month prior to the doctor’s appointment. Of the 138 subjects, 54 respondents (39%) reported skipping at least one dose of medication in the month prior to follow-up visit. A statistically significant relationship was observed between variables such as age and the existence of comorbidities and adherence with treatment recommendations. Patients aged >65 years less frequently missed taking medication doses compared to those aged ≤65 years (*p* < 0.011). Patients suffering at least one comorbid disease better adhered to treatment instructions than those with only CML (*p* < 0.011) (Table 2). There was no correlation between gender (*p* = 0.237), education (*p* = 0.121), place of residence (*p* = 0.460), family circumstances (*p* = 0.387), and adherence with treatment recommendations. Neither was there any link between adverse events from TKI with patients missing their medication doses (*p* = 0.843) (Table 2). There were no differences in adherence among patients treated with imatinib, dasatinib and nilotinib (*p* = 0.659).

**Table 2:** Analysis of factors potentially influencing the risk of skipping TKI doses in the month prior to doctor’s appointment (*n* = 138)

Variable	Numbers of patients reporting skipping TKI medication doses (*n* = 54)	Numbers of patients, not reporting skipping TKI medication doses (*n* = 84)	*P* value	OR [95% CI]
Gender				
Women	30 (55.6)	38 (45.2)	0.237	–
Men	24 (44.4)	46 (54.8)		
Age				
≤65 years	46 (85.2)	55 (65.5)	0.011	0.57 [0.37–0.89]
>65 years	8 (14.8)	29 (34.5)		
Education				
Basic	12 (22.2)	31 (36.9)	0.121	–
Secondary	27 (50.0)	29 (34.5)		
Higher	15 (27.8)	24 (28.6)		
Place of residence				
Countryside	22 (40.7)	29 (34.5)	0.460	–
Town/city	32 (59.3)	55 (65.5)		
Family circumstances				
Single	10 (18.5)	11 (13.1)	0.387	–
Living with family	44 (81.5)	73 (86.9)		
Comorbidities				
None	27 (50.0)	24 (28.6)	0.011	0.40 [0.20–0.82]
At least 1	27 (50.0)	60 (71.4)		
Adverse effects				
None	13 (24.1)	19 (22.6)	0.843	–
At least 1	41 (75.9)	65 (77.4)		

Within the *n* = 140 study group, two subjects did not reply as to whether they skipped medication doses in the month prior to the doctor’s appointment

None of tested potential predictors including age and presence of comorbid disease significantly influenced the probability of adherence in multivariate logistic regression analysis (data not shown).

### Skipping TKI doses throughout the treatment period

Of the 140 subjects, 72 (51.4%) admitted to skipping such doses from the start of their treatment to the time at which our assessment was performed. Patients with secondary education more frequently missed medication doses compared to those with higher and primary education (*p* = 0.014). There were no significant differences in adherence between patients’ age (*p* = 0.437), gender (*p* = 0.310), place of residence (*p* = 0.185), family circumstances (*p* = 0.750), the presence of comorbidities (*p* = 0.094) nor adverse drug effects (*p* = 0.682) (Table 3). There were no differences in adherence among patients treated with imatinib, dasatinib and nilotinib (*p* = 0.249).

**Table 3:** Analysis of factors potentially influencing the risk of skipping TKI doses throughout the treatment period (*n* = 140)

Variable	Numbers of patients reporting skipping TKI medication doses (*n* = 72)	Numbers of patients, not reporting skipping TKI medication doses (*n* = 68)	*P* value	OR [95% CI]
Gender				
Women	39 (54.2)	31 (45.6)	0.310	–
Men	33 (45.8)	37 (54.4)		
Age				
≤65 years	55 (76.4)	48 (70.6)	0.437	–
>65 years	17 (23.6)	20 (29.4)		
Education				
Basic	16 (22.2)	27 (39.7)	0.014	
Secondary	38 (52.8)	20 (29.4)		0.31 [0.14–0.71]
Higher	18 (25.0)	21 (30.9)		0.69 [0.29–1.67]
Place of residence				
Countryside	30 (41.7)	21 (30.9)	0.185	–
Town/city	42 (58.3)	47 (69.1)		
Family circumstances				
Single	12 (16.7)	10 (14.7)	0.750	–
Living with family	60 (83.3)	58 (85.3)		
Comorbidities				
None	31 (43.1)	20 (29.4)	0.094	–
At least 1	41 (56.9)	48 (70.6)		
Adverse effects				
None	18 (25.0)	15 (22.1)	0.682	–
At least 1	54 (75.0)	53 (77.9)		

None of tested potential predictors including education significantly influenced the probability of adherence in multivariate logistic regression analysis (data not shown).

### Relationship between skipping TKI doses and duration of therapy

With increased treatment duration, the number of patients missing their medication doses significantly rose in the month prior to their follow-up visit as well as throughout their whole treatment. The longer the TKI dose regimen lasted, then the lower was the self-assessment of adherence. The data are shown in Table 4.

**Table 4:** Relationship between duration of TKI therapy with self-assessed skipping of recommended medication doses

Duration of therapy	Number of patients reporting skipping TKI doses *n* = 54	Number of patients, not reporting skipping TKI doses *n* = 84	*P* value	OR [95% CI]
Skipping doses within the month prior to follow-up visit				
<1 year	2 (3.7%)	24 (28.6%)	<0.001	
≥1–<2 years	7 (13.0%)	15 (17.9%)		5.6 [1.0–30.6]
≥2 years	45 (83.3%)	45 (83.3%)		12.0 [2.7–53.8]

Duration of therapy	Number of patients reporting skipping TKI doses *n* = 72	Number of patients, not reporting skipping TKI doses *n* = 68	*P* value	OR [95% PU]
Skipping doses within the entire duration of treatment				
<1 year	4 (5.6%)	22 (32.4%)	<0.001	
≥1–<2 years	8 (11.1%)	14 (20.6%)		3.1 [0.8–12.4]
≥2 years	60 (83.3%)	32 (47.0%)		10.3 [3.3–32.5]

In addition, the odds ratio for adherence was tested throughout the treatment period. Those who took medication for over 2 years were 12.17 times more likely to skip their doses than those taking the drug for less than 1 year.

### Reasons why TKI doses were skipped

Among the 72 patients who missed taking TKI doses throughout their treatment, 38 (52.8%) did so unintentionally (e.g. due to forgetfulness, distraction). Others, however, 34 (47.2%), did this consciously. The most common reasons were: being away for several days (29.4%), antibiotic-treated infection (23.5%), medically unrelated to the primary disease—e.g. previous operations, food poisoning coupled with strong vomiting, sore throat preventing swallowing (17.6%), TKI side effects (17.6%), no prescription (11.8%), social meetings (11.8%), worry over TKI side effects (5.9%), “medication holiday” (2.9%). In those who reported repeatedly missing their medication, the same persons would provide several reasons (as survey allowed for more than one reply per question).

When analysing the reported TKI side effects, the most common were gastrological symptoms (41.4%), various kinds of pain (40.7%), oedema (37.9%), psychological symptoms (32.1%) and others (25.7%); more than one side effect was permitted to be chosen.

### Information received from the doctor

Sufficiently adequate instructions for adherence with medication regimens were acknowledged by 131 (93.6%) subjects concerning any given TKI treatment. Eight other subjects claimed this information to be inadequate whilst one declared of not been informed at all. All of the aforementioned 131 subjects declared that they knew when to take TKIs in relation to mealtimes and were also aware that consuming any products containing St. John’s Wort was forbidden. Three subjects (2.1%) were unaware that eating grapefruit or its processed products had also been banned.

### Patient’s self-assessment of their treatment adherence

Among 54 patients reported skipping at least one dose of medication in the month prior to follow-up doctor’s appointment, 34 (63.0%) felt that they ‘always’ followed instructions and 17 (31.5%) ‘almost always’. Two patients (3.7%) followed instructions “from time to time” and 1 (1.8%) “never” followed instructions.

In the 72 subjects admitting some non-adherence throughout the treatment period, 50 (69.4%) considered that in fact they ‘always’ followed instructions, whilst 17 (23.6%) patients did so ‘almost always’. Two patients (2.8%) followed instructions “from time to time”, and 3 subjects did not respond to question.

Patients aged >65 years significantly better evaluated their adherence compared to patients aged <30 years (88.6 vs. 42.9%; *p* < 0.030).

## Discussion

Our study revealed that a significant proportion of the surveyed CML patients treated with TKI was found not to adhere to treatment recommendations. Half reported skipping at least one drug dose throughout their treatment period and 39% did so within the last month. In addition, patient’s self-assessment overestimated adherence; around 60% of those that missed at least one dose of medication within the last month and admitting some non-adherence throughout the treatment period, considered that they ‘always’ follow instructions.

Our results indicated that medication doses were inadvertently omitted by 54% subjects, with reasons commonly given as being forgetfulness. It is therefore vital to introduce techniques for jogging the memory, where Moon et al. [25] observed that patients with chronic phase CML who received daily text reminders to take IM observed better adherence than patients without such assistance.

The problem of non-adherence to medical treatment has been fairly well documented in the literature, but primarily relates to non-cancerous chronic diseases. AIDS patients skipping doses have been associated with worse outcomes where average ‘adherence’ rates have been observed at 49.5% [26]. Munger et al. [27] demonstrated that only 59% of hypertensive patients comply with therapeutic instructions. Similar adherence rates are seen in patients suffering from depression (40–70%) [28], asthma (50%) [29], osteoporosis (50%) [30] and Parkinson’s disease (65%) [31]. Doesch et al. [32] found that 30–70% of heart transplant patients regularly do not take their medication.

With the spread in oral anti-cancer therapies, the problem of low ‘adherence’ also increasingly applies to cancer patients [16, 33]. Livaudais et al. [34] surveyed adherence rates when hormone therapy was initiated for 13,753 women with breast cancer treated in 1996–2007. This found that only 70% of the patients took their prescriptions and started therapy. Similar results were observed by Kimmick et al. [35], where for those women who started therapy, 20% discontinued treatment after 1 year whilst 40% took less than the prescribed medication doses. Furthermore, Partridge et al. [36] showed a decrease in adherence for women with breast cancer receiving hormonal drugs during 4 years of follow-up.

Most adherence studies on haematological patients have dealt with CML patients treated with TKI. A prospective and observational study, ADAGIO, (Adherence Assessment with Glivec: Indicators and Outcomes) covered 202 CML patients treated with IM [24]. It was found that only 14.2% patients showed 100% adherence. Seventy per cent of patients took less than that prescribed (down to 29% less) but 14.8% took more (up to 202%).

It is still not clear which factors affecting adherence are the most important. The results of different studies have brought conflicting results. An important factor bearing influence on adherence seems to be the duration of therapy. Our study showed that adherence significantly decreased over time. Missing medication within the month prior the visit in the first 2 years of therapy was admitted by 11.1% of subjects and after 2 years this became 88.9% (*p* < 0.001). Similarly, over the whole treatment period, rates of skipping medication were correspondingly 11.2% in the first 2 years of treatment and 88.8% after 2 years TKI treatment (*p* < 0.001). A study by Partridge et al. [37] on a group of 2378 women with breast cancer treated with tamoxifen in 1990–1996 found that overall adherence was 77% in the first year of treatment but fell to 50% in the fourth year. Similar results were reported by Barron et al. [38], where 78% of postmenopausal women with breast cancer after a year of treatment were taking medication, whilst only 65% did so after 3 years. In CML patients treated with TKI, Efficace et al. [39] demonstrated that the shorter the therapy time the higher the adherence. In contrast the aforementioned ADAGIO study did not confirm these conclusions [24].

Our study revealed that the presence of at least one coexisting disease was linked with favourable adherence. The similar results were presented in ADAGIO study [24]. Also in the study of Efficace et al. [39] a multivariate analyses showed that the taking of additional medication for comorbidities was a factor benefitting adherence. It seems likely that patients already accustomed to taking more drugs show better adherence, as they have been educated to be systematic in taking their medication.

We observed that patients aged >65 years more rarely omit taking their TKI doses. Indeed, older age has been identified as factor favourably affecting adherence in other studies. Marin et al. [22] demonstrated a strong correlation between patient age and treatment adherence. It was observed that more frequently, younger subjects did not follow the instructions and that median-aged patients of 43 years showed a less than 90% adherence compared with adherence rates exceeding 90% in people aged over 53 years. In contrast, Noens et al. [24] in ADAGIO study found older age as a factor negatively influenced adherence.

Our results indicated that adverse effects of TKI did not significantly affect adherence to treatment recommendations, although 18% subjects did indeed give adverse events as the cause for skipping their medication dose. Similarly the ADAGIO study did not show any significant relationship between IM adverse effects with patient’s treatment adherence [24]. In contrast Marin et al. [22] noted that side effects from taking IM were linked with worse adherence, these being bone and joint pain, asthenia, nausea and muscle spasms. A study by Jabbour et al. [40] found that the side effects of drugs are the most common reason for reducing patients’ doses or interrupting the therapeutic process. Also Marin et al. [41] found that intolerance to the drug was one of the reasons why some patients discontinued therapy. It is therefore important for the doctor to make the patient aware of the drug’s possible side effects and to enquire about adverse events at each visit so as to take action as and when appropriate. Richardson et al. [42] demonstrated that educational programs which inform about the disease and any adverse medication reactions so arising are associated with a prolonged survival of haematological patients.

We did not find any differences in adherence among patients treated with imatinib, dasatinib and nilotinib. There are conflicting findings with respect to differences in adherence and persistence with dasatinib and nilotinib during CML treatment. Trivedi et al. [43] found that dasatinib patients had significantly higher adherence and lower discontinuation rates compared with patients receiving second-line nilotinib. In contrast Guérin et al. [44] revealed that among patients treated in the second-line CML setting, those treated with nilotinib had significantly higher adherence compared to patients treated with dasatinib. It must be underlined that in our study small number of patients in dasatinib and especially nilotinib group influenced the value of the results, which need the confirmation in the larger cohort.

## Limitations

Some limitations of our study have to be considered. First, our sample is relatively small, especially dasatinib and nilotinib group and larger studies are still needed to confirm the results. Second, in our study we used survey method which is one most frequently adopted for evaluating adherence. The limitation of this method is that although anonymous one can not exclude reporting bias; where subjects’ replies are intended to please the surveyor. Moreover, because the survey is voluntary, one can assume that patients wished to cooperate with the doctor and thus are psychologically more inclined to comply with the prescribed treatment regimen [36, 45].

## Conclusions

Our study confirmed that failure to adhere to treatment recommendations in CML patients is very common and should therefore be routinely assessed by means of clinical interview. Moreover, we found that more than 60% of the subjects who reported skipping TKI doses admitted that they ‘always’ followed therapeutic recommendations. This observation underlines the important role of medical staff in raising patient awareness about the importance of their adherence. Our results confirm that low treatment adherence in CML patients should always be taken into account as a cause of treatment failure, which should be considered by physicians before taking the decision to introduce next generation TKI therapy.
